# Abnormal differentiation of Sandhoff disease model mouse-derived multipotent stem cells toward a neural lineage

**DOI:** 10.1371/journal.pone.0178978

**Published:** 2017-06-02

**Authors:** Yasuhiro Ogawa, Katsutoshi Kaizu, Yusuke Yanagi, Subaru Takada, Hitoshi Sakuraba, Kazuhiko Oishi

**Affiliations:** 1 Department of Pharmacology, Meiji Pharmaceutical University, Tokyo, Japan; 2 Department of Clinical Genetics, Meiji Pharmaceutical University, Tokyo, Japan; Instituto Butantan, BRAZIL

## Abstract

In Sandhoff disease (SD), the activity of the lysosomal hydrolytic enzyme, β-hexosaminidase (Hex), is lost due to a *Hexb* gene defect, which results in the abnormal accumulation of the substrate, GM2 ganglioside (GM2), in neuronal cells, causing neuronal loss, microglial activation, and astrogliosis. We established induced pluripotent stem cells from the cells of SD mice (SD-iPSCs). In the present study, we investigated the occurrence of abnormal differentiation and development of a neural lineage in the asymptomatic phase of SD *in vitro* using SD mouse fetus-derived neural stem cells (NSCs) and SD-iPSCs. It was assumed that the number of SD mouse fetal brain-derived NSCs was reduced and differentiation was promoted, resulting in the inhibition of differentiation into neurons and enhancement of differentiation into astrocytes. The number of SD-iPSC-derived NSCs was also reduced, suggesting that the differentiation of NSCs was promoted, resulting in the inhibition of differentiation into neurons and enhancement of that into astrocytes. This abnormal differentiation of SD-iPSCs toward a neural lineage was reduced by the glucosylceramide synthase inhibitor, miglustat. Furthermore, abnormal differentiation toward a neural lineage was reduced in SD-iPSCs with *Hexb* gene transfection. Therefore, differentiation ability along the time axis appears to be altered in SD mice in which the differentiation ability of NSCs is promoted and differentiation into neurons is completed earlier, while the timing of differentiation into astrocytes is accelerated. These results clarified that the abnormal differentiation of SD-iPSCs toward a neural lineage *in vitro* was shown to reflect the pathology of SD.

## Introduction

In Sandhoff disease (SD), the *Hexb* gene encoding the β-subunit of the lysosomal hydrolytic enzyme, β-hexosaminidase consisting of Hex A and Hex B subunits, is mutated and the enzyme activities of both subunits are simultaneously lost, resulting in an inability to hydrolyze GM2 ganglioside and the accumulation of GM2 mainly in neurons. The disease occurs in infancy or early childhood and progressive neuropathy develops, such as mental retardation, motor impairments, and convulsion. However, the mechanisms responsible for GM2 accumulation-induced cranial nervous system impairments have not yet been elucidated in detail, and one reason for this is the absence of information on the timing of neural lineage development in patients.

*Hexb* gene-knockout (*Hexb*^−/−^) mice (SD mice) are a mouse model of the pathology of SD [1]. In SD mice, no apparent symptoms are observed until approximately 8 weeks after birth, when movement gradually becomes slow and limb tremors appear. Motor impairments, such as a startle response, ataxia, and muscle weakness, appear approximately 12 weeks after birth, and these mice die at approximately 16 weeks old. The molecular-pathological mechanisms underlying SD remain unclear; however, based on previous studies using SD mice, the following hypothesis is generally accepted: the breakage of intracellular structures and cell death due to the accumulation of GM2 ganglioside and metabolites trigger symptoms 8 weeks after birth, and inflammatory and autoimmune systems involving microglia and astrocytes are activated with the progression of symptoms, inducing apoptosis in neurons and neurodegeneration [2, 3]. Although changes that occur during the period from ontogeny to the asymptomatic phase have not yet been clarified, abnormal neurites of neurons prepared from the hippocampal region of SD mouse fetuses [4] and nerve cell death in the dorsal root ganglion prepared from 4-week-old SD mice in the asymptomatic phase [5] have been reported. These findings suggest that changes in neurons in the embryonic stage and asymptomatic phase are the partial cause of subsequent cranial nervous system dysfunction. However, in order to elucidate the intrinsic cause, the changes that occur before the development of symptoms need to be reproduced *in vitro*.

We established induced pluripotent stem cells (iPSCs) from SD mice (SD-iPSCs), which is a mouse model that reflects the pathology of SD. Using the stromal cell-derived inducing activity (SDIA) method [6], the differentiation of SD-iPSCs toward a neural lineage was induced and investigated. The ability of SD-iPSCs to differentiate into neural stem cells (NSCs) and that of NSCs to differentiate into neurons were markedly weaker than those of wild-type mouse-derived iPSCs (WT-iPSCs) [7]. This reduction in differentiation ability was partially normalized by the transfection of the *Hexb* gene in SD-iPSCs, clarifying that the *Hexb* gene defect reduced the ability to differentiate into neurons. Therefore, the following events have been suggested to occur over time in SD mice: the abnormal differentiation and development of NSCs in the embryonic stage ⇒ an increase in activated astrocytes and activation of immune system cells, such as microglia, in the asymptomatic phase ⇒ activation of the inflammatory and autoimmune systems ⇒ neurodegeneration and nerve cell death. In the present study, we clarified the occurrence of abnormal differentiation and development of a neural lineage in the asymptomatic phase of SD *in vitro* using SD mouse fetus-derived NSCs and SD-iPSCs.

Mammalian NSC culture methods including that for human cells have been established, and long-term cultures are possible in the presence of growth factors, such as basic fibroblast growth factor (bFGF) [8–11]. Cultured NSCs may be induced to differentiate into neurons and glia cells by removing growth factors. Therefore, the self-replication ability and pluripotency of NSCs may be reproduced *in vitro*. In order to investigate the abnormal neural differentiation of SD mouse fetus-derived NSCs, NSCs from the fetal brains of SD mice and heterozygous litter mates at embryonic day 12.5 were cultured, and NSC properties, proliferation ability, and differentiation ability toward a neural lineage were compared.

The abnormal differentiation of SD-iPSCs toward a neural lineage was investigated in an analysis employing the SDIA method and serum-free floating culture of embryoid body-like aggregates with quick reaggregation (SFEBq), which is capable of inducing the differentiation of cerebral cortex-like nerve tissue [12]: The expression intensities of the NSC markers, nestin and Sox2, were compared between SD-iPSC-derived NSCs prepared by the 2 neural lineage differentiation methods in order to investigate their properties as NSCs. Their proliferative abilities were also analyzed based on the size and number of neurospheres. Furthermore, whether neurons and glia cells have abnormal differentiation abilities was examined using immunostaining. In order to clarify whether the changes observed were directly induced by the *Hexb* gene defect, the recovery of the lost ability by the transfection of the *Hexb* gene in SD-iPSCs (HEXB-iPSCs) was studied.

The glucosylceramide synthase inhibitor, miglustat, is a drug that has been approved to treat lysosomal diseases, such as type 1 Gaucher disease and Niemann-Pick disease type C, by substrate inhibition therapy. The normalization of changes induced by miglustat was investigated. The *in vitro* differentiation system of SD-iPSCs toward a neural lineage may be utilized to screen for compounds that act on abnormal differentiation and the development of a neural lineage, i.e., the effects of compounds may be investigated based on the number and size of iPSC-derived NSC colonies formed on PA6 stroma cells using the SDIA method and also on the size and shape of embryoid bodies formed by iPSC-derived NSCs using SFEBq.

## Materials and methods

### Mouse models

All animal procedures were performed in accordance with the Guidelines for Animal Experimentation by the Japanese Association for Laboratory Animal Science, and were approved by the Institutional Animal Use and Care Committee of Meiji Pharmaceutical University. *Hexb*^−/−^ mice (C57BL/6 × 129sv) were kindly provided by Dr. Richard L. Proia (Genetics of Development and Disease Branch, National Institute of Diabetes, and Digestive and Kidney Disease, National Institutes of Health, Bethesda, MD, USA). The mice were bred by mating them with C57BL/6 mice (Japan SLC, Hamamatsu, Japan) and were maintained under specific pathogen-free conditions in the animal facilities at Meiji Pharmaceutical University. Genotyping of these mice was performed by PCR as described previously [13].

### Isolation and propagation of NSCs

An NSC culture was performed as described previously [14]. Pregnant mice were euthanized with avertin. Briefly, embryos (embryonic day 12.5) were removed and placed on a Petri dish containing Hanks’ balanced salt solution (HBSS). After decapitation, the brain was removed. The cortex was dissected out and carefully triturated with a fire-polished Pasteur pipette in N2 medium (DMEM/F12 plus N2 supplements) in the presence of 20 ng/ml bFGF and 2 μg/ml heparin. Cells were collected by centrifugation and resuspended in N2 medium. Viable cells were plated on 60-mm culture plastic dishes (Corning, NY) at 10^5^ cells/ml in N2 medium, and were maintained at 37°C in 5% CO_2_/ 95% air. The number of primary neurospheres generated was assessed 4 days after plating. Spheres were collected by centrifugation for 5 min at 450 x *g* and dissociated mechanically to single-cell suspensions. Some cells were processed for immunocytochemistry and the remainder were replated at 10^5^ cells/ml in fresh N2 medium with bFGF. By 4 days after plating, secondary neurospheres with the ability to undergo further passages had formed.

### Differentiation of NSCs

Spheres were dissociated mechanically to single-cell suspensions and replated onto poly-ornithine/fibronectin-coated 48-well culture plates (Corning, NY) at 6.5 x 10^4^ cells/cm^2^. One hr after plating, the differentiation of NSCs was induced by replacing bFGF-containing medium with fresh N2 medium supplemented with 1% fetal bovine serum (FBS) and culturing for 3 days.

### iPS cell culture

SD-iPSCs (SFM1022)[7] and WT-iPSCs (iPS-MEF-Ng-20D-17; purchased from the Riken Cell Bank) were maintained on mitomycin C-treated MEFs in DME medium (Wako Co., Tokyo, Japan) containing 15% FBS (Invitrogen), 2 mM L-glutamine, 0.1 mM non-essential amino acids, 0.1 mM 2-mercaptoethanol, 50 U/ml penicillin, and 50 μg/ml streptomycin as described elsewhere [7].

### Transfection

In order to construct an oriP/EBNA1-based episomal plasmid (pEB-HexB-HA-Neo), HA-tagged HexB (MGC collection #100015010, Invitrogen) generated by PCR with a 5’ primer containing a SalI site and a 3’ primer containing a NotI site was ligated into the XhoI/NotI site of pEBMulti-Neo (Wako). The pEB-HexB-HA-Neo plasmid was electroporated using the pulse generator, Cuy21Pro-vitro (NEPA Gene, Chiba, Japan), according to the manufacturer’s protocol. Transfected cells were selected with 500 μg/mL G418.

### Induction of neural differentiation

In order to achieve differentiation using the SDIA method, dissociated SD-iPSCs were co-cultured on PA6 stromal cells (purchased from the Riken Cell Bank) as single cells at 10^4^ cells/60-mm dish or 5 x 10^2^ cells/48-well culture plate to form colonies in Glasgow’s modification of Eagles medium (G-MEM) containing 10% knockout serum replacement (KSR), 2 mM glutamine, 1 mM pyruvate, 0.1 mM non-essential amino acids, and 0.1 mM 2-mercaptoethanol, and were then cultured for 7 days at 37°C with 5% CO_2_, as described previously [7]. In order to induce primary neurospheres, iPSC colonies were detached by trypsinization, mechanically dissociated into single cells, and then cultured for several days in N2 medium containing 2% B27 supplement minus vitamin A and 20 ng/mL bFGF.

Regarding differentiation using SFEBq, dissociated SD-iPSCs were plated on 96-well low cell-adhesion plates (PrimeSurface 96U, MS-9096U, Sumitomo Bakelite Co., Ltd., Tokyo, Japan) in G-MEM supplemented with 7% KSR at a density of 4500 cells/well and cultured for 7 days at 37°C with 5% CO_2_, as described previously [15]. After 7 days of culturing, the aggregates were transferred to a bacterial-grade Petri dish in N2 medium and cultured for a further 5 days. In order to induce primary neurospheres, iPSC aggregates were mechanically dissociated into single cells, and then cultured for several days in N2 medium containing 2% B27 supplement minus vitamin A and 20 ng/mL bFGF.

### Immunostaining

Immunostaining was performed as described previously [7]. Fluorescence images were obtained under an Axio Imager M2 using an AxioCam MRm digital camera (Carl Zeiss, Jena, Germany). The primary antibodies used were as follows: anti-nestin (Rat-401; DSHB), anti-Sox2 (Millipore, Bedford, MA), anti-neuronal class III β-tubulin (Tuj1; Covance, Richmond, CA), anti-glial fibrillary acidic protein (GFAP; Dako, Carpinteria, CA), and anti-HA.11 (16B12; Covance, Denver, PA). The secondary antibodies used were an Alexa Fluor 488 anti-rabbit IgG antibody, Alexa Fluor 488 anti-mouse IgG antibody, Alexa Fluor 488 anti-chick IgG antibody, Alexa Fluor 568 anti-rabbit IgG antibody, Alexa Fluor 568 anti-mouse IgG antibody, Alexa Fluor 568 anti-chick IgG antibody, and Alexa Fluor 647 anti-rabbit IgG antibody (all purchased from Molecular Probes, Life Technologies, Eugene, OR).

### EdU proliferation assay

Dissociated single-cell suspensions were plated onto poly-ornithine/fibronectin-coated 48-well culture plates (Corning, NY), and were maintained to allow adhesion at 37°C in 5% CO_2_/ 95% air. One hour after plating, proliferating NSCs were determined by using the Click-iT EdU Alexa Fluor 488 Imaging kit (Invitrogen, Carlsbad, CA) according to the manufacturer’s protocol. Briefly, cells were incubated with 10 μM EdU for 1h before fixation, permeabilization, and EdU staining. Cell nuclei were stained with DAPI. Immunostaining for nestin was performed as described in the Immunostaining protocol. For fetal brain-derived NSCs, 10 fluorescence images of different fields (1.3 × 1.8 mm) were obtained using a fluorescence microscope (Axio Imager. M2, Carl Zeiss, Jena, Germany). The percentages of EdU/nestin double-positive cells (% of total nestin-positive count) were then determined using ImageJ (National Institutes of Health, Bethesda, MD). For iPSC-derived NSCs, EdU/nestin double-positive cells were counted using the IN Cell Analyzer 2200 (GE Healthcare). An average of nine images per well were collected using a 10× objective. Data analysis was performed using the IN Cell Developer Toolbox software.

### Statistical analysis

Values are expressed as the means ± standard error (S.E.). The significance of differences between experimental groups was analyzed using the unpaired Student’s *t*-test (StatView for Mac).

## Results

### Neurons and astrocytes increased in SD mouse fetal brain-derived neurospheres

**Fig 1:**
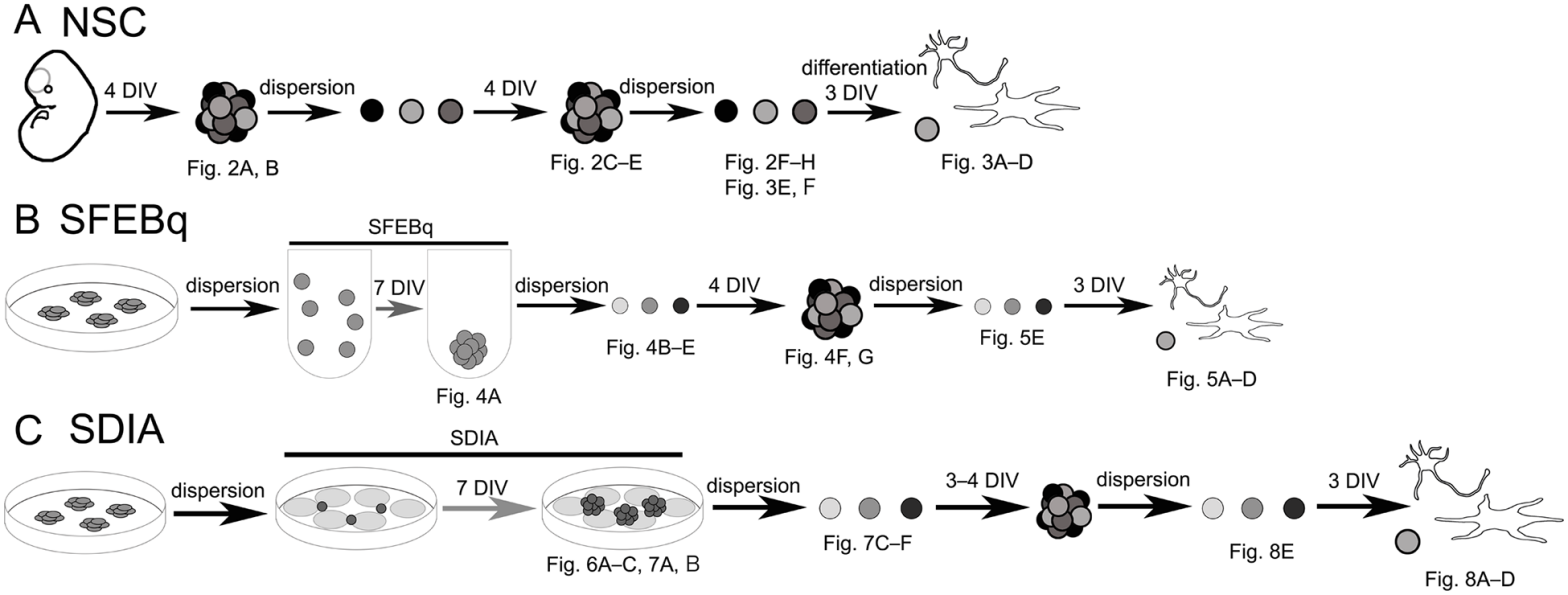
Schematic diagram of the experiment. (A) Isolation, propagation, and differentiation of mouse fetal brain-derived NSCs. (B) Induction of neural differentiation using SFEBq method. (C) Induction of of neural differentiation using SDIA method. The figure data obtained at corresponding stages were indicated in the scheme.

**Fig 2:**
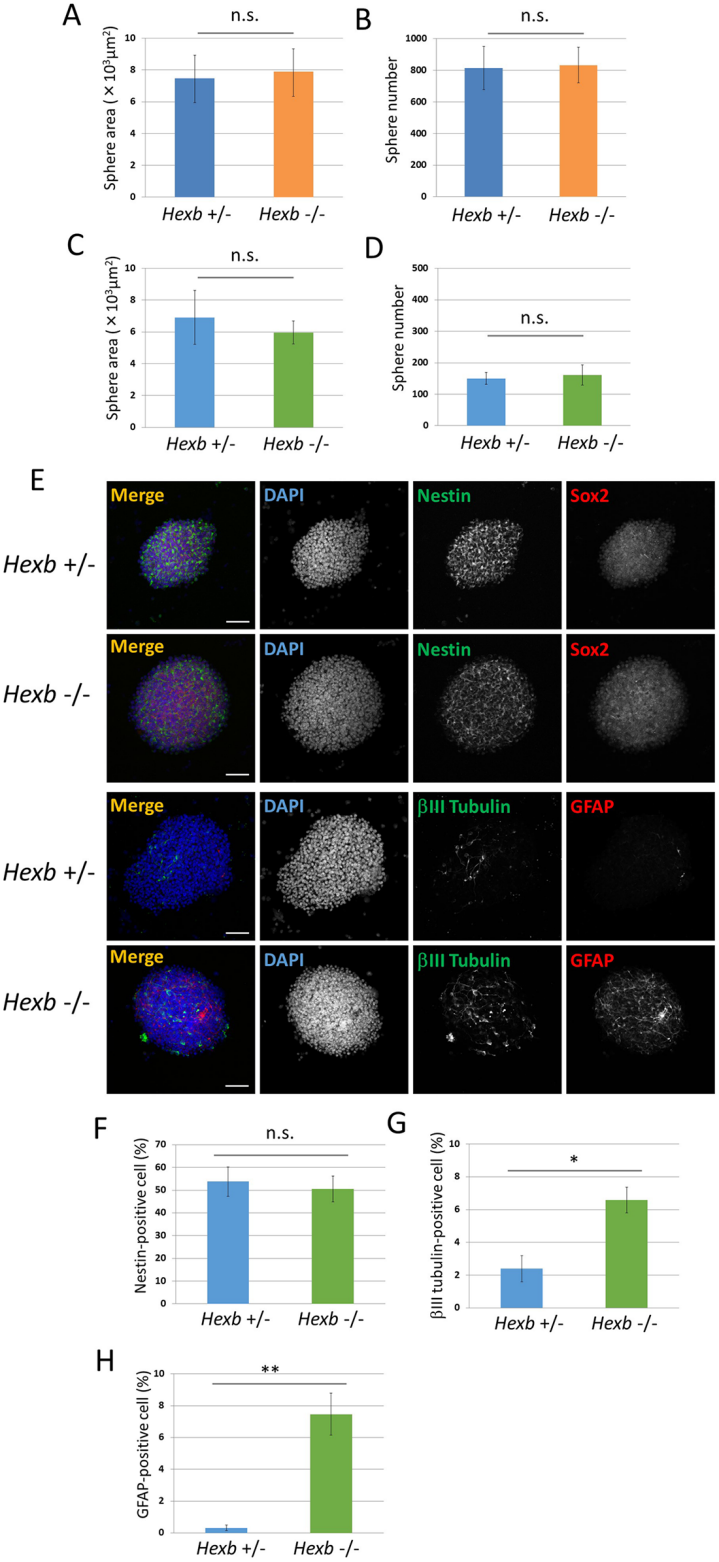
Characterization of NSCs derived from the fetal brain of the mouse SD model. (A−D) Characterization of neurospheres isolated from cerebral cortices of *Hexb*^+/−^ and *Hexb*^−/−^ mice at embryonic day 12.5. For each sample, 20 images of different fields (1.3 × 1.8 mm) were photographed, and the areas (A, C) and numbers (B, D) of primary (A, B) and secondary (C, D) neurospheres cultured for 4 days were measured. (E−H) Expression pattern of primary neurospheres isolated from cerebral cortices at embryonic day 12.5 of *Hexb*^+/−^ and *Hexb*^−/−^ mice. (E) Primary neurospheres were collected on poly-L-ornithine/fibronectin-coated glass slides by cytospin centrifugation, fixed, and immunostained for nestin (green), Sox2 (red), βIII tubulin (green), and GFAP (red). Blue represents DAPI staining. The scale bar indicates 100 μm. (F−H) Primary neurospheres were dissociated mechanically to single-cell suspensions. Cells were plated onto poly-L-ornithine/fibronectin-coated glass coverslips. One hour after plating, cells were fixed and immunostained for nestin, βIII tubulin, and GFAP with DAPI nuclear staining. For each sample, 20 fluorescence images of different fields (1.3 × 1.8 mm) were obtained. The percentages of positive cells (% of total DAPI count) were then determined using ImageJ (National Institutes of Health, Bethesda, MD). The percentages of NSCs (F), neurons (G), and astrocytes (H) were measured. Values represent the mean ± S.E. of five independent experiments. n.s.: Not significantly different (*P* > 0.05), *P<0.05, **P<0.01.

NSCs isolated and cultured from the mouse fetal brain grew forming neurospheres. The SD mouse (*Hexb*^−/−^) fetal brain was minced and cultured for 4 days, and the size of the neurospheres that formed were photographed and the area that they occupied was analyzed using ImageJ (Fig 1A). Cells obtained from *Hexb*^+/-^ fetal brain serve as normal controls[5, 16–21]. The areas of neurospheres were 7.48 × 10^3^ ± 1.44 × 10^3^ μm^2^ in *Hexb*^+/−^ mice and 7.89 × 10^3^ ± 1.12 × 10^3^ μm^2^ in *Hexb*^−/−^ mice, showing no significant difference (Fig 2A). Differences in the numbers of neurospheres were microscopically analyzed; there were 819 ± 136 neurospheres in *Hexb*^+/−^ mice and 832 ± 112 in *Hexb*^−/−^ mice, showing no significant difference (Fig 2B).

Second-generation neurospheres prepared by dispersion and a 4-day re-culture of neurospheres were similarly analyzed. The areas of neurospheres were 6.91 × 10^3^ ± 1.69 × 10^3^ μm^2^ in *Hexb*^+/−^ mice and 5.96 × 10^3^ ± 0.73 × 10^3^ μm^2^ in *Hexb*^−/−^ mice (Fig 2C), and their numbers were 150.3 ± 18.7 and 161 ± 31.7, respectively (Fig 2D), showing no significant differences. Neurospheres were dispersed and immunostained using antibodies against the NSC marker, nestin, neuron marker, βIII tubulin, and astrocyte marker, GFAP (Fig 2E). The percentage of nestin-positive NSCs contained in neurospheres were 53.8 ± 6.45 and 50.6 ± 5.68% in *Hexb*^+/−^ and *Hexb*^−/−^ mice, respectively, showing no significant difference (Fig 2F). In contrast, the percentage of βIII tubulin-positive neurons were 2.40 ± 0.80 and 6.58 ± 0.78%, respectively, showing that the efficiency of neurons was significantly higher in *Hexb*^−/−^ mice than in *Hexb*^+/−^ mice (Fig 2G). The percentage of GFAP-positive astrocytes were 0.32 ± 0.17 and 7.46 ± 1.32%, respectively, showing that the efficiency was significantly higher in *Hexb*^−/−^ mice than in *Hexb*^+/−^ mice (Fig 2H). These results suggest that differentiation from NSCs was promoted in *Hexb*^−/−^ mouse fetal brain-derived neurospheres, increasing neurons and astrocytes.

### When the differentiation of SD mouse fetal brain-derived NSCs was induced, NSCs and neurons decreased and astrocytes increased

**Fig 3:**
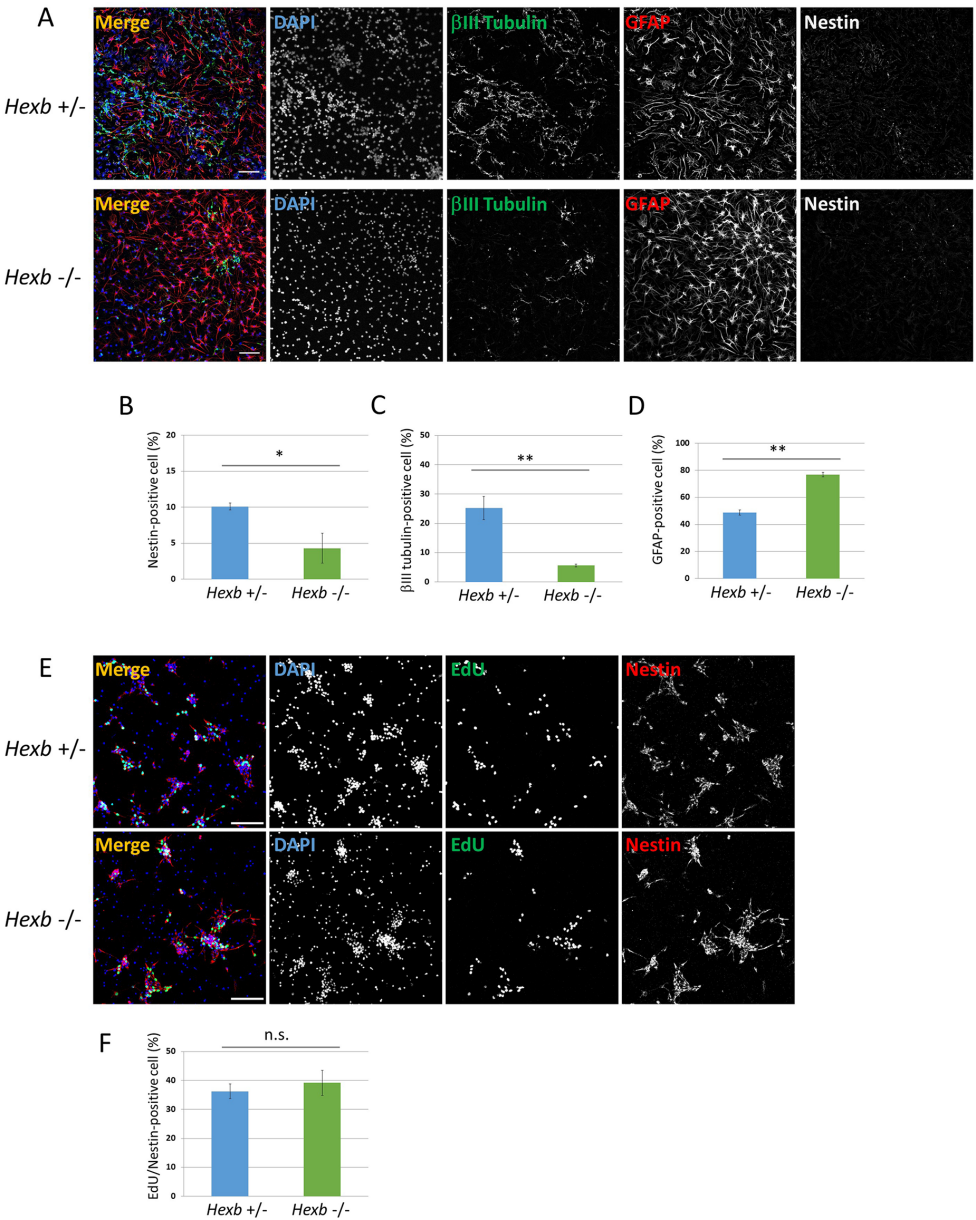
Neural differentiation from NSCs isolated from cerebral cortices at embryonic day 12.5 of *Hexb*^+/−^ and *Hexb*^−/−^ mice. Primary neurospheres isolated from the cerebral cortices at embryonic day 12.5 of *Hexb*^+/−^ and *Hexb*^−/−^ mice were dissociated mechanically to single-cell suspensions and replated onto poly-ornithine/fibronectin-coated culture dishes. One hour after plating, the differentiation of NSCs was induced by replacing bFGF-containing medium with fresh N2 medium supplemented with 1% FBS and cultured for 3 days. (A) Immunostaining of differentiated cells for βIII tubulin (green), GFAP (red), and nestin (white) with DAPI nuclear staining (blue). The scale bar indicates 100 μm. (B−D) For each sample, 20 fluorescence images of different fields (1.3 × 1.8 mm) were obtained. The percentages (% of total DAPI count) of NSCs (B), neurons (C), and astrocytes (D) were evaluated. Values represent the mean ± S.E. of five independent experiments. (E) One hour after plating, proliferating NSCs were determined by using the Click-iT EdU Alexa Fluor 488 Imaging kit (green). Immunostaining of differentiated cells for nestin (red) with DAPI nuclear staining (blue). (F) For each sample, 10 fluorescence images of different fields (1.3 × 1.8 mm) were obtained. The percentages of EdU/nestin double-positive cells (% of total nestin-positive count) were evaluated. Values represent the mean ± S.E. of six independent experiments. n.s.: Not significantly different (*P* > 0.05), *P<0.05, **P<0.01.

*Hexb*^−/−^ mouse-derived neurospheres were dispersed, followed by a 3-day adhesion culture to induce differentiation and then immunostaining using antibodies against nestin, βIII tubulin, and GFAP (Figs 1A and 3A). The percentage of NSCs after the induction of differentiation were 10.1 ± 0.48 and 4.8 ± 2.08% in *Hexb*^+/−^ and *Hexb*^−/−^ mice, respectively, showing that the efficiency was significantly lower in *Hexb*^−/−^ mice than in *Hexb*^+/−^ mice (Fig 3B).

The percentage of neurons were 25.2 ± 3.97 and 5.6 ± 0.49%, respectively, showing that the efficiency was significantly lower in *Hexb*^−/−^ mice than in *Hexb*^+/−^ mice (Fig 3C). In contrast, the percentage of astrocytes were 48.8 ± 1.87 and 76.8 ± 1.61%, respectively, showing that the efficiency was significantly higher in *Hexb*^−/−^ mice than in *Hexb*^+/−^ mice (Fig 3D). The percentages of EdU/nestin double-positive cells per nestin-positive cells were 36.2 ± 2.51 and 39.1 ± 4.33 in *Hexb*^+/−^ and *Hexb*^−/−^ mice, respectively, showing no significant difference (Fig 3E and 3F). These results indicate that the differentiation from NSCs was promoted, thereby inhibiting differentiation into neurons and increasing astrocytes.

### NSCs were unchanged, whereas neurons and astrocytes increased in cerebral cortex-like tissue prepared from SD-iPSCs

**Fig 4:**
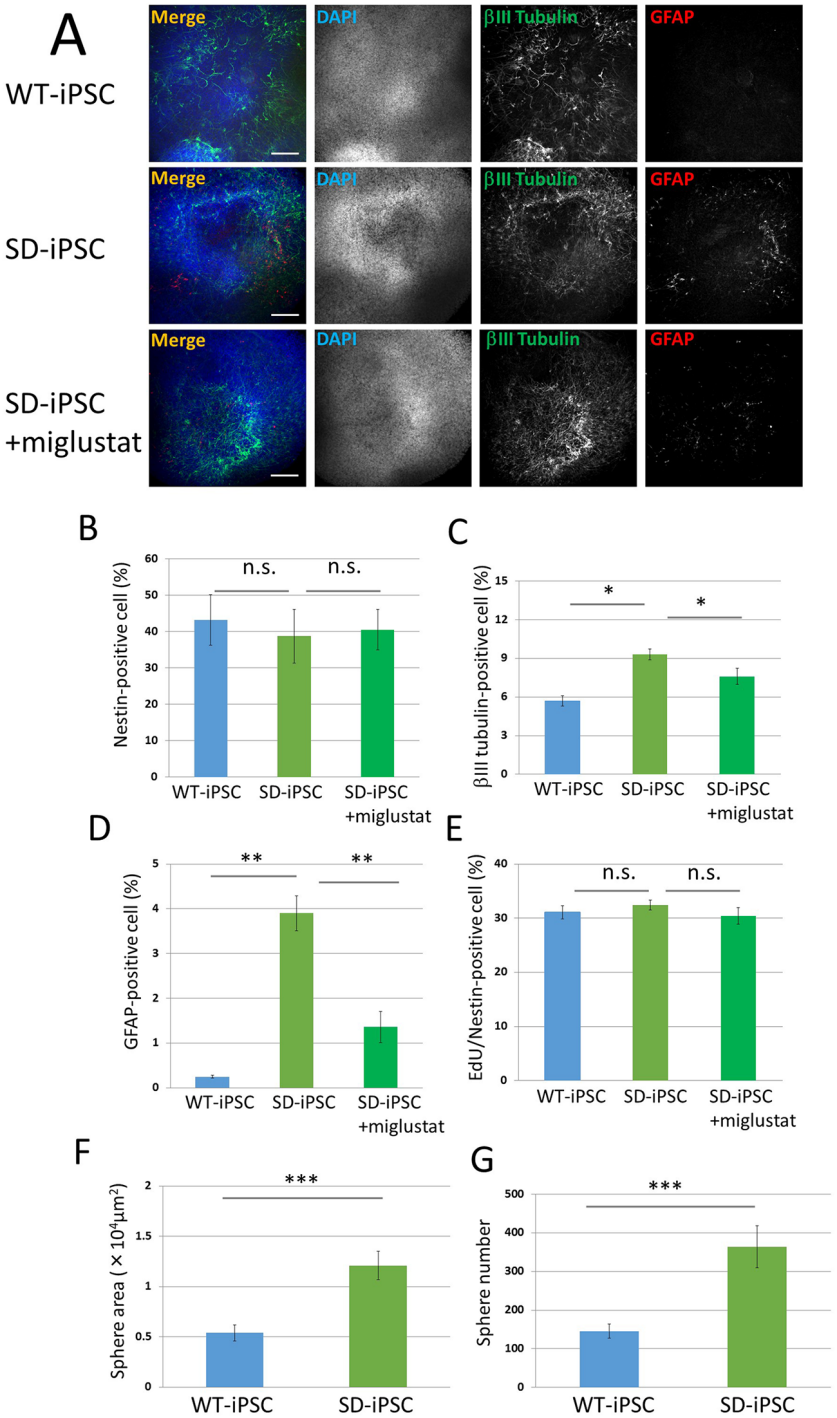
Characterization of SFEBq-induced cerebral cortical tissues and neurospheres isolated from SFEBq-induced cortical tissues of WT-iPSC and SD-iPSC. Dissociated WT-iPSCs and SD-iPSCs were plated on 96-well low cell-adhesion plates in G-MEM medium supplemented with 7% KSR in the presence or absence of 5 μM miglustat, an inhibitor of the enzyme glucosylceramide synthase, and cultured for 7 days. (A) Immunostaining of WT-iPSC and SD-iPSC (with or without miglustat) cortical tissues for βIII tubulin (green) and GFAP (red) with DAPI nuclear staining (blue). The scale bar indicates 100 μm. (B−E) Cortical tissues were dissociated mechanically to single-cell suspensions. Cells were plated onto poly-L-ornithine/fibronectin-coated glass coverslips. One hour after plating, the cells were fixed and immunostained for nestin, βIII tubulin, and GFAP with DAPI nuclear staining (B-D). For each sample, 20 fluorescence images of different fields (1.3 × 1.8 mm) were obtained. The percentages (% of total DAPI count) of NSCs (B), neurons (C), and astrocytes (D) were evaluated. (E) One hour after plating, proliferating NSCs were determined by using the Click-iT EdU Alexa Fluor 488 Imaging kit (green) (S1 Fig). EdU/nestin double-positive cells were counted using the IN Cell Analyzer 2200. (F and G) Neurospheres isolated from SFEBq-induced cerebral cortical tissues of WT-iPSC and SD-iPSC. For each sample, 20 images of different fields (1.3 × 1.8 mm) were photographed, and the areas (F) and numbers (G) of primary neurospheres cultured for 4 days were measured. Values represent the mean ± S.E. of five independent experiments. n.s.: Not significantly different (*P* > 0.05), *P<0.05, **P<0.01, ***P<0.005.

Cerebral cortex-like tissue prepared by a 7-day culture using SFEBq was dispersed and immunostained using antibodies against nestin, βIII tubulin, and GFAP (Figs 1B and 4A). The percentage of nestin-positive NSCs were 43.2 ± 6.92 and 38.7 ± 7.41% in wild-type and SD cerebral cortex-like tissues, respectively, showing that the efficiency of NSCs contained in SD-iPSC-derived tissue was not significantly different from that in WT-iPSC-derived tissue (Fig 4B). In contrast, the percentage of βIII tubulin-positive neurons were 5.7 ± 0.41 and 9.3 ± 0.42% in wild-type and SD tissues, respectively, showing that the efficiency was significantly higher in SD than in wild-type tissue (Fig 4C). The percentage of GFAP-positive astrocytes were 0.25 ± 0.03 and 3.90 ± 0.39%, respectively, showing that the efficiency was significantly higher in SD than in wild-type tissue (Fig 4D). When miglustat (5 μM) was added during the preparation of cerebral cortex-like tissue with SD-iPSCs, the percentage of astrocytes was 1.36 ± 0.35%, showing that the increase in astrocytes was inhibited. The percentages of EdU/nestin double-positive cells in SFEBq-induced cerebral cortical tissues of WT-iPSC and SD-iPSC were 31.1 ± 1.21 and 32.4 ± 0.91%, respectively, showing no significant difference (Fig 4E). When miglustat (5 μM) was added during the preparation of cerebral cortex-like tissue with SD-iPSCs, the percentages of EdU/nestin double-positive cells was 30.4 ± 1.57%, showing no effect. These results suggest that differentiation from NSCs was promoted in cerebral cortex-like tissue prepared from SD-iPSCs, as observed in SD mouse fetal brain-derived neurospheres, decreasing NSCs and increasing neurons and astrocytes.

### The proliferation ability of NSCs cultured from SD-iPSC-derived cerebral cortex-like tissue was not changed

**Fig 5:**
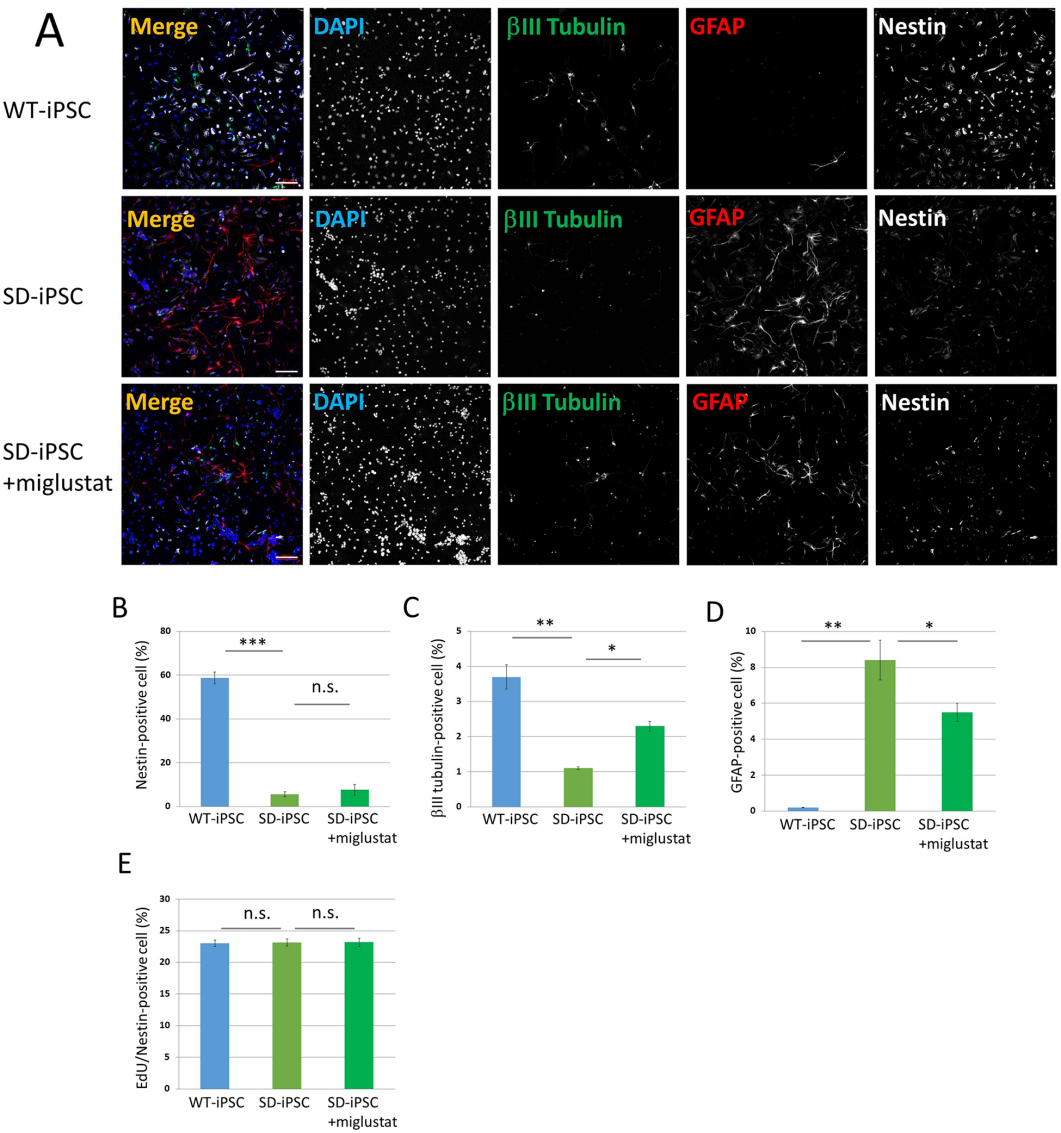
Neural differentiation from NSCs isolated from SFEBq-induced cerebral cortical tissues of WT-iPSC and SD-iPSC. Primary neurospheres isolated from the SFEBq-induced cerebral cortical tissues of WT-iPSC and SD-iPSC (with or without miglustat) were dissociated mechanically to single-cell suspensions and replated onto poly-ornithine/fibronectin-coated culture dishes. One hour after plating, the differentiation of NSCs was induced by replacing bFGF-containing medium with fresh N2 medium supplemented with 1% FBS and culturing for 3 days. (A) Immunostaining of differentiated cells for βIII tubulin (green), GFAP (red), and nestin (white) with DAPI nuclear staining (blue). The scale bar indicates 100 μm. (B−D) For each sample, 20 fluorescence images of different fields (1.3 × 1.8 mm) were obtained. The percentages (% of total DAPI count) of NSCs (B), neurons (C), and astrocytes (D) were evaluated. (E) One hour after plating, proliferating NSCs were determined by using the Click-iT EdU Alexa Fluor 488 Imaging kit (green) (S2 Fig). EdU/nestin double-positive cells were counted using the IN Cell Analyzer 2200. Values represent the mean ± S.E. of five independent experiments. n.s.: Not significantly different (*P* > 0.05), *P<0.05, **P<0.01, ***P<0.005.

Cerebral cortex-like tissue prepared by a 7-day culture employing SFEBq was dispersed and cultured for 4 days. The neurospheres that formed were photographed and their area was analyzed using ImageJ (Fig 1B). The area was 0.54 × 10^4^ ± 0.08 × 10^4^ μm^2^ in wild-type tissue and 1.21 × 10^4^ ± 0.14 × 10^4^ μm^2^ in SD tissue, showing that the efficiency significantly increased in SD (Fig 4F). The number of neurospheres was then analyzed microscopically in order to investigate whether differences existed between the tissue types. The numbers of neurospheres were 146 ± 18.3 and 364 ± 54.1, respectively, showing a significantly higher number in SD tissue (Fig 4G). The percentages of EdU/nestin double-positive cells in neurospheres of WT-iPSC and SD-iPSC were 23.0 ± 0.52 and 23.1 ± 0.59%, respectively, showing no significant difference (Fig 5E). When miglustat (5 μM) was added during the preparation of cerebral cortex-like tissue with SD-iPSCs, the percentages of EdU/nestin double-positive cells was 23.2 ± 0.69%, showing no effect. The results obtained also clarified that the proliferation ability of NSCs cultured from SD-iPSC-derived cerebral cortex-like tissue was not changed.

### When the differentiation of NSCs cultured from SD-iPSC-derived cerebral cortex-like tissue was induced, NSCs and neurons decreased and astrocytes increased

Neurospheres cultured from SD-iPSC-derived cerebral cortex-like tissue were dispersed followed by a 3-day adhesion culture to induce differentiation (Fig 1B). Nestin, βIII tubulin, and GFAP were immunostained using antibodies (Fig 5A). The percentage of NSCs after the induction of differentiation were 58.7 ± 2.71 and 5.6 ± 1.05% in wild-type and SD tissues, respectively, showing that the efficiency was significantly lower in SD than in wild-type tissue (Fig 5B). The percentage of neurons were 3.70 ± 0.35 and 1.10 ± 0.04%, respectively, showing a significantly lower efficiency in SD tissue (Fig 5C). In contrast, the percentage of astrocytes were 0.21 ± 0.02 and 8.42 ± 1.28%, respectively, showing that the efficiency was significantly higher in SD-iPSCs (Fig 5D). When miglustat (5 μM) was added during the preparation of cerebral cortex-like tissue from SD-iPSCs, the decrease observed in the efficiency of neurons and increase in the efficiency of astrocytes were partially normalized. The results obtained clarified that differentiation from NSCs was promoted and differentiation into neurons was inhibited, thereby increasing astrocytes.

### The efficacy of NSC colony formation on PA6 stroma cells was low in SD-iPSCs

**Fig 6:**
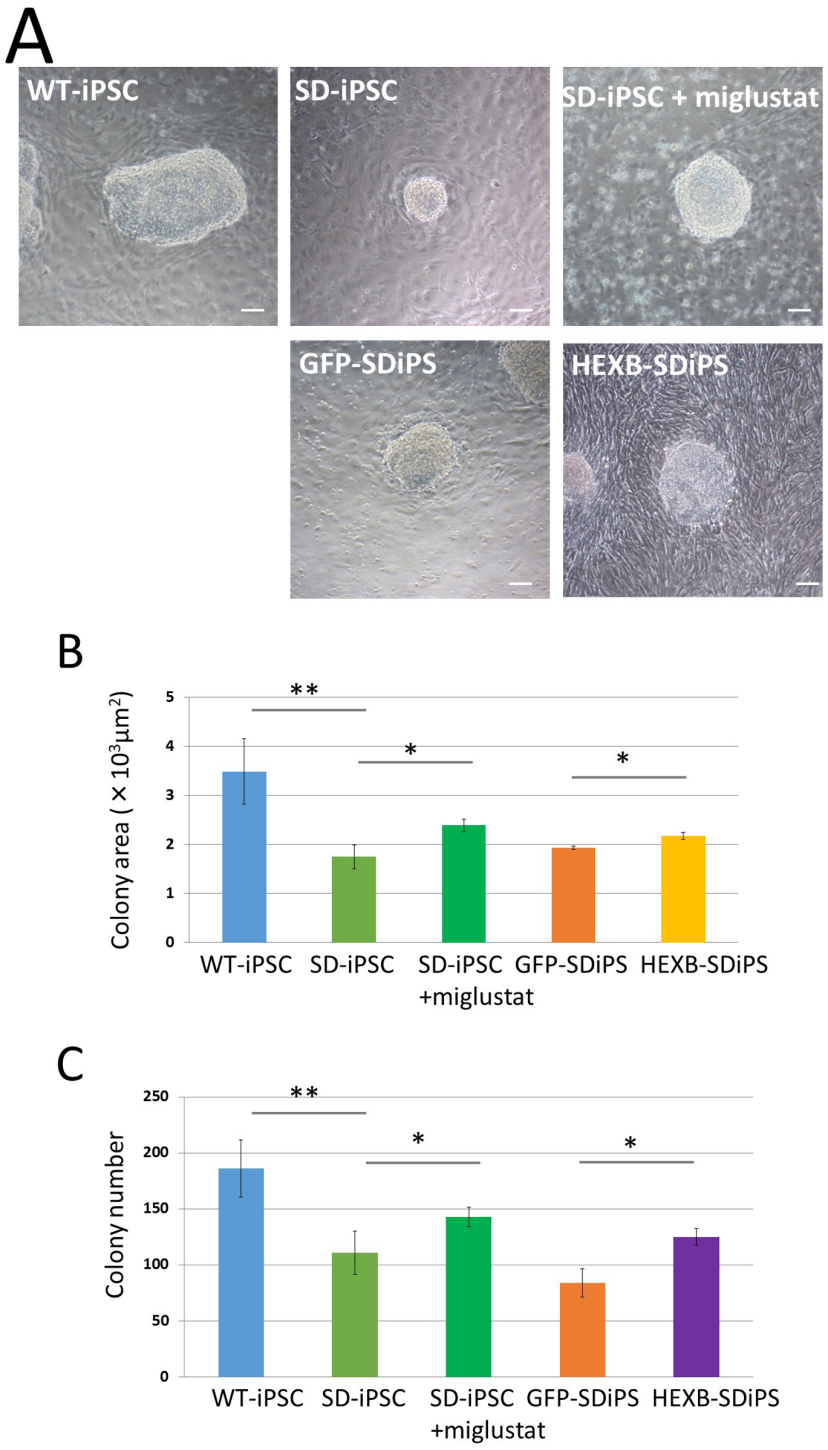
Characterization of SDIA-induced colonies at day 7 of differentiation. Dissociated WT-iPSCs, SD-iPSCs, GFP-SD-iPSCs (GFP-SDiPS), and HEXB-SD-iPSCs (HEXB-SDiPS) were co-cultured on PA6 stromal cells as single cells to form colonies in G-MEM medium supplemented with 10% KSR in the presence or absence of 5μM miglustat, and cultured for 7 days. (A) Phase-contrast image of WT-iPSC, SD-iPSC (with or without miglustat), GFP-SD-iPSC, and HEXB-SD-iPSC colonies. The scale bar indicates 100 μm. (B and C) For each sample, 20 images of different fields (1.3 × 1.8 mm) were photographed, and the areas (B) and numbers (C, as a function of the initial cell number plated) of SDIA-induced colonies at day 7 of differentiation were evaluated. Values represent the mean ± S.E. of five independent experiments. *P<0.05, **P<0.01.

When iPSCs are cultured on PA6 stroma cells, colonies containing a large number of NSCs are formed (Figs 1C and 6A). In order to investigate the size of NSC colonies formed by a 7-day co-culture, the NSC colonies formed were photographed and the area that they occupied was analyzed using ImageJ. Their areas were 3.49 × 10^3^ ± 0.67 ×10^3^ μm^2^ in wild-type tissue and 1.76 × 10^3^ ± 0.24 × 10^3^ μm^2^ in SD tissue, showing a significantly smaller area in SD (Fig 6B). The presence of a difference in the number of NSC colonies was then microscopically investigated. Their numbers were 186 ± 25.4 and 111 ± 19.2 in wild-type and SD tissues, respectively, showing a significant decrease in SD (Fig 6C). When miglustat (5 μM) was added to a co-culture of SD-iPSCs and PA6 stroma cells, the decreases observed in the colony area and count were significantly inhibited (Fig 6B and 6C). In HEXB-SD-iPSCs prepared by the transfection of the *Hexb* gene in SD-iPSCs, the NSC colony area and count were significantly higher than in those prepared from GFP-SD-iPSCs prepared by *EGFP* gene transfection in SD-iPSCs (Fig 6B and 6C).

### NSCs and neurons decreased and astrocytes increased in SD-iPSC-derived NSC colonies formed on PA6 stroma cells

**Fig 7:**
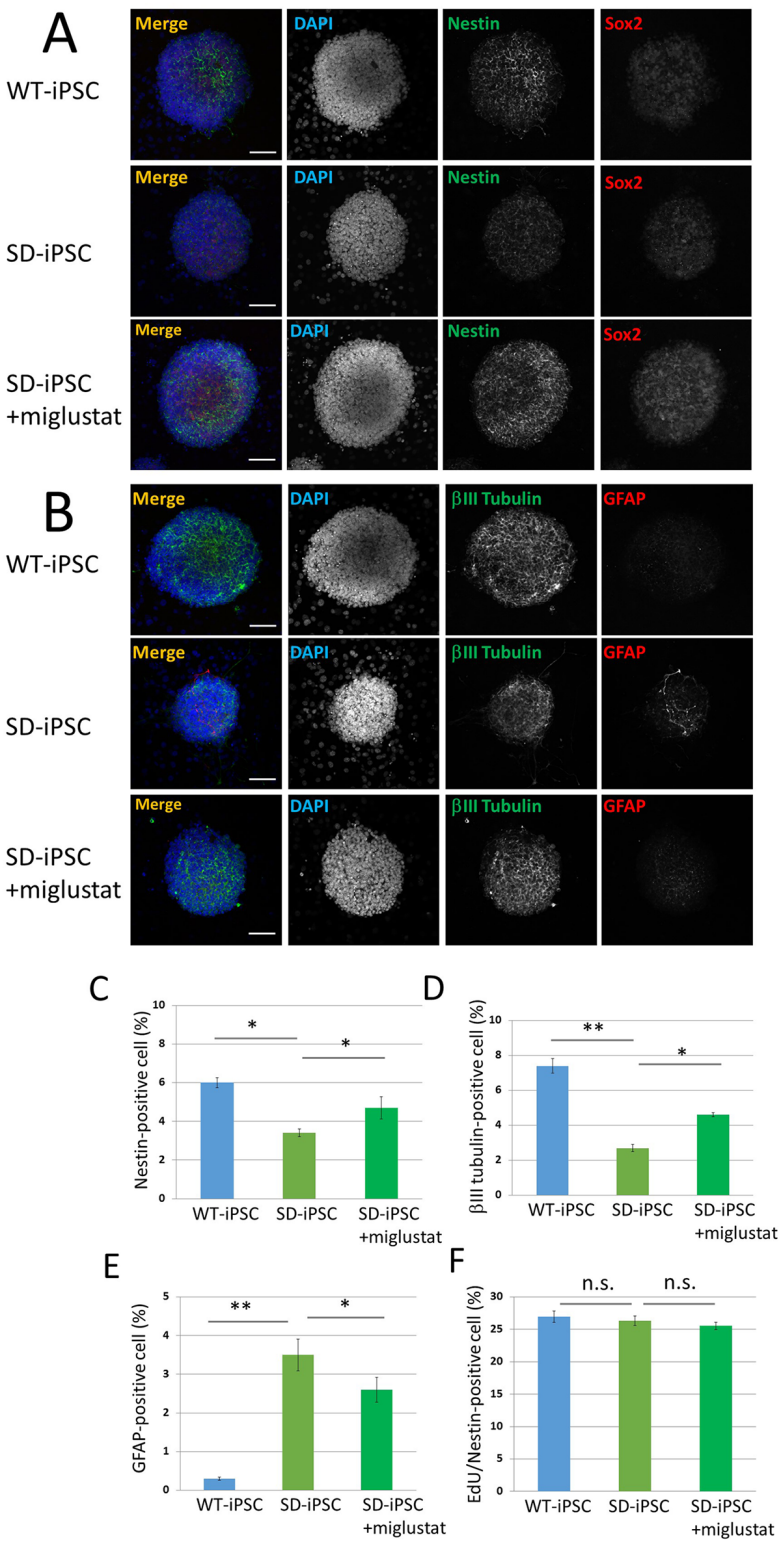
Immunocytochemical characterization of SDIA-induced colonies. (A and B) SDIA-induced WT-iPSC and SD-iPSC (with or without miglustat) colonies were fixed, and immunostained for nestin (green), Sox2 (red), βIII tubulin (green), and GFAP (red). Blue represents DAPI staining. The scale bar indicates 100 μm. (C−E) SDIA-induced colonies were dissociated mechanically to single-cell suspensions. The cells were plated onto poly-L-ornithine/fibronectin-coated glass coverslips. One hour after plating, the cells were fixed and immunostained for nestin, βIII tubulin, and GFAP with DAPI nuclear staining. For each sample, 20 fluorescence images of different fields (1.3 × 1.8 mm) were obtained. The percentages (% of total DAPI count) of NSCs (C), neurons (D), and astrocytes (E) were evaluated. (F) One hour after plating, proliferating NSCs were determined by using the Click-iT EdU Alexa Fluor 488 Imaging kit (green) (S3 Fig). EdU/nestin double-positive cells were counted using the IN Cell Analyzer 2200. Values represent the mean ± S.E. of five independent experiments. Not significantly different (*P* > 0.05), *P<0.05, **P<0.01.

NSC colonies prepared by a 7-day culture employing the SDIA method (Fig 7A and 7B)　were dispersed and immunostained using antibodies against nestin, Sox2, βIII tubulin, and astrocyte GFAP. The percentage of nestin-positive NSCs were 6.00 ± 0.27 and 3.40 ± 0.20% in wild-type and SD tissues, respectively, showing that the efficiency was significantly lower in SD than in wild-type tissue (Fig 7C). When miglustat (5 μM) was added during a co-culture of SD-iPSCs on PA6 stroma cells, the percentage of NSCs was 4.70 ± 0.58%, reducing the decrease in NSCs. The percentage of βIII tubulin-positive neurons were 7.41 ± 0.42 and 2.72 ± 0.22%, respectively, showing that the efficiency was significantly lower in SD than in wild-type tissue (Fig 7D). When miglustat (5 μM) was added, the percentage of neurons was 4.6 ± 0.12%, showing that the decrease in neurons was reduced. In contrast, the percentage of GFAP-positive astrocytes were 0.30 ± 0.04 and 3.53 ± 0.41%, respectively, showing that the efficiency was significantly higher in SD than in wild-type tissues (Fig 7E). The percentage of astrocytes was 2.60 ± 0.32% when miglustat (5 μM) was added, showing that the increase in astrocytes was reduced. The percentages of EdU/nestin double-positive cells in SDIA-induced colonies of WT-iPSC and SD-iPSC were 27.0 ± 0.08 and 26.3 ± 0.75%, respectively, showing no significant difference (Fig 7F). When miglustat (5 μM) was added during the preparation of cerebral cortex-like tissue with SD-iPSCs, the percentages of EdU/nestin double-positive cells was 25.5 ± 0.52%, showing no effect. The results obtained clarified that differentiation from NSCs was promoted in NSC colonies prepared from SD-iPSCs, as observed in SD mouse fetal brain-derived neurospheres, which may have decreased NSCs and increased neurons and astrocytes.

### When the differentiation of NSCs cultured from SD-iPSC-derived NSC colonies was induced, NSCs and neurons decreased and astrocytes increased

**Fig 8:**
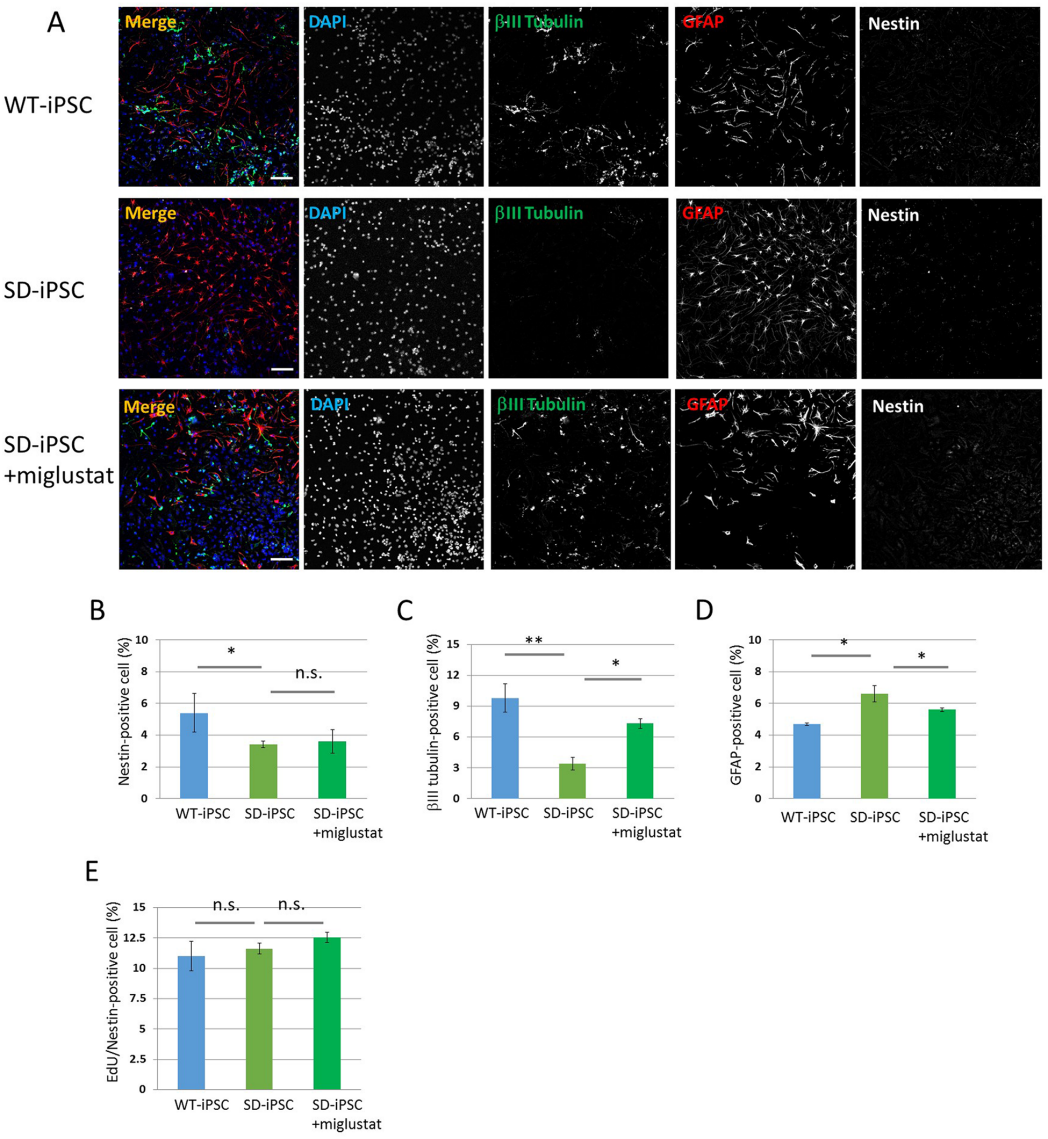
Neural differentiation from NSCs isolated from SDIA-induced colonies. Primary neurospheres isolated from the SDIA-induced colonies of WT-iPSC and SD-iPSC (with or without miglustat) were dissociated mechanically to single-cell suspensions and replated onto poly-ornithine/fibronectin-coated culture dishes. One hour after plating, the differentiation of NSCs was induced by replacing bFGF-containing medium with fresh N2 medium supplemented with 1% FBS and culturing for 3 days. (A) Immunostaining of differentiated cells for βIII tubulin (green), GFAP (red), and nestin (white) with DAPI nuclear staining (blue). The scale bar indicates 100 μm. (B−D) For each sample, 20 fluorescence images of different fields (1.3 × 1.8 mm) were obtained. The percentages (% of total DAPI count) of NSCs (B), neurons (C), and astrocytes (D) were evaluated. (E) One hour after plating, proliferating NSCs were determined by using the Click-iT EdU Alexa Fluor 488 Imaging kit (green) (S4 Fig). EdU/nestin double-positive cells were counted using the IN Cell Analyzer 2200. Values represent the mean ± S.E. of five independent experiments. n.s.: Not significantly different (*P* > 0.05), *P<0.05, **P<0.01.

Neurospheres cultured from SD-iPSC-derived NSC colonies were dispersed followed by a 3-day adhesion culture to induce differentiation and immunostaining using antibodies against nestin, βIII tubulin, and GFAP (Figs 1C and 8A). The rates of NSCs after the induction of differentiation were 5.41 ± 1.22 and 3.40 ± 0.21% in wild-type and SD colonies, respectively, showing that the efficiency was significantly lower in SD than in wild-type colonies (Fig 8B). The percentage of neurons were 9.8 ± 1.39 and 3.4 ± 0.62%, respectively, showing that the efficiency was significantly lower in SD than in wild-type colonies (Fig 8C). In contrast, the percentage of astrocytes were 4.70 ± 0.08 and 6.60 ± 0.51%, respectively, showing that the efficiency was significantly higher in SD than in wild-type colonies (Fig 8D). When miglustat (5 μM) was added during the preparation of NSC colonies from SD-iPSCs, the decrease observed in the efficiency of neurons and increase in the efficiency of astrocytes were reduced. The percentages of EdU/nestin double-positive cells in SDIA-induced neurospheres of WT-iPSC and SD-iPSC were 11.0 ± 1.22 and 11.6 ± 0.45%, respectively, showing no significant difference (Fig 8E). When miglustat (5 μM) was added during the preparation of cerebral cortex-like tissue with SD-iPSCs, the percentages of EdU/nestin double-positive cells was 12.6 ± 0.43%, showing no effect. The results obtained clarified that differentiation from NSCs was promoted, which inhibited differentiation into neurons and increased astrocytes.

## Discussion

The development of the central nervous system begins with neural induction at the time of gastrulation. The mesoderm contacts the presumptive ectoderm due to gastrulation, which induces the neural plate in the median region of the ectoderm, and this subsequently becomes hollow and forms a neural tube comprising neuroepithelial cells. The neural tube progresses along the anteroposterior and dorsoventral axes and develops into complex structures that individually have different characteristics, ultimately forming the brain and spinal cord[22, 23].

In this developmental process, marked changes also occur at the cell level. Neuroepithelial cells are undifferentiated NSCs, and new NSCs are generated by the repeated symmetrical division of these cells. This period corresponds to embryonic day 8 to 10 in the development of the mouse central nervous system. NSCs start to asymmetrically divide from approximately embryonic day 11.5, generating new NSCs, and the other daughter cells generate neuronal precursor cells that are destined to become neurons. Neuronal precursor cells repeat symmetrical division and produce neurons. This neurogenesis continues until the late embryonic stage, and they start to produce glial cells, such as astrocytes and oligodendrocytes, at approximately embryonic day 17.5. The differentiation ability of NSCs is strictly controlled temporary and spatially, and this is one of the factors influencing the arrangement of various types of neurons and glia cells at appropriate locations and times.

NSCs were cultured from the SD mouse (*Hexb*^−/−^) fetal brain at embryonic day 12.5, and their properties, proliferation ability, and differentiation ability toward a neural lineage were investigated. In SD mouse fetal brain-derived neurospheres, NSCs decreased, and neurons and astrocytes increased. When the differentiation of SD mouse fetal brain-derived NSCs was induced, NSCs and neurons decreased and astrocytes increased. A previous study reported that NSCs originate from radial glial cells derived from neuroepithelial cells [24], and they have pluripotency to differentiate into neurons, astrocytes, and oligodendrocytes, constituting a neural lineage. After embryonic day 17.5, they do not produce neurons, and start to produce astrocytes. Control by Notch signaling is considered to be a cause of this cell differentiation switching following the time axis [25–27]. Notch is a molecule that is expressed by undifferentiated cells. Its intracellular domain is cut and transfers into the nucleus in response to a stimulation by the Notch ligand expressed on adjacent cells, and forms the RBP-J/Notch complex. This complex induces the expression of the basic-helix-loop-helix (bHLH)-type transcription inhibitors, Hes1 and Hes5. Notch ligand expression periodically increases and decreases (oscillation), and the resulting Hes1 oscillation inhibits differentiation and maintains stem cells [28, 29]. In the late developmental stage, Notch signaling induces differentiation into astrocytes through the phosphorylation of STAT3 [30]. The STAT3 binding site in the astrocyte-specific gene promoter is methylated in the early developmental stage, and the ability to differentiate into astrocytes is acquired through demethylation in the late developmental stage [31]. Regarding signals of differentiation into neurons, relationships with bHLH-type transcription factors, such as neurogenin and Mash1, have been reported [32–35]. The transcription of these transcription factors for induction of neural lineage commitment is inhibited by Hes1 and Hes5, as described above. Mash1 is known to not only induce differentiation into neurons, but also actively inhibits differentiation into glia cells [35].

Accordingly, cell differentiation ability following this time axis is altered in SD mice: the number of NSCs is assumed to decrease, thereby completing differentiation in neurons earlier, while the timing of differentiation in astrocytes is accelerated. In neurogenesis, neural lineage differentiation is controlled temporary and spatially, and various types of neurons and glia cells are arranged at appropriate locations and times. One possibility is that the phenotype of SD mice affects the essential developmental program and leads to neurological dysfunction.

In the method to induce neuronal differentiation from mouse ES/iPS cells, an embryoid body is generally prepared in a suspension culture in the presence of serum. The differentiation of three germ layers may be induced in this embryoid body, similar to the actual embryo, and the promotion of differentiation toward a neural lineage in the presence of retinoic acid has been reported [36]. However, this induction method simultaneously induces differentiation into the endoderm and mesoderm. Moreover, the addition of retinoic acid simultaneously induces caudalization of induced neural tissues [37]. Thus, difficulties are associated with the induction of rostral nerve tissue including the telencephalon.

A large number of studies on the methods to induce neuronal differentiation from mouse ES cells were subsequently performed, and SFEB was established, in which mouse ES cells cultured in suspension in serum-free medium without growth factors, such as retinoic acid and bFGF, and feeder cells form cell aggregates, through which neuronal differentiation is efficiently induced [38]. Nerve cells induced by SFEBq are mainly those in the telencephalon and retain reactivity with the reported region-determining factors, similar to that *in vivo*. A large number of cerebral cortex precursor cells may be induced in a culture using a U-bottomed 96-well plate (SFEBq) [12]. A three-dimensional culture using SFEBq not only induces a large number of cerebral cortex precursor cells, but also forms the neuroepithelial structure required for the development of central nervous system tissue and produces cerebral cortex tissue with a laminar structure. Thus, the abnormality of SD in the developmental stage may now be observed in a state close to the development of the cerebrum.

When NSCs were cultured from the SD mouse fetal brain at embryonic day 12.5 and their properties, proliferation ability, and differentiation ability toward a neural lineage were investigated, the number of the NSCs decreased and the ability to differentiate into neurons were significantly weaker, whereas the ability to differentiate into astrocytes was significantly stronger in SD mice than in heterozygous mice. Thus, we cultured SD-iPSCs using the SFEBq method and prepared cerebral cortex-like tissue. Similar to previous findings on SD mouse fetus-derived NSCs, the number of the NSCs decreased and the ability to differentiate into neurons of NSCs was significantly reduced, while the ability to differentiate into astrocytes was significantly increased. Therefore, it was assumed that the differentiation from NSCs in SD-iPSC-derived cerebral cortex-like tissue was promoted, as observed in SD mouse fetal brain-derived neurospheres, resulting in the inhibition of differentiation into neurons and enhancement of that into astrocytes.

Miglustat suppresses the synthesis of ganglioside by inhibiting glucosylceramide synthase, which acts on the first step of glycosphingolipid synthesis. Since downstream GM2 ganglioside synthesis is also inhibited, the drug is expected to reduce the accumulation of GM2 ganglioside. Thus, we investigated the reversal of changes observed using SFEBq by miglustat. Miglustat significantly restored the changes induced in SD-iPSCs. The results obtained clarified that NSCs induced from SD-iPSCs employing SFEBq had similar properties to those of NSCs prepared from SD mouse fetus-derived NSCs and reflected abnormal differentiation toward a neural lineage. Miglustat partially normalized abnormal differentiation toward a neural lineage by reducing the accumulation of GM2 ganglioside, which demonstrated that the abnormal differentiation observed *in vitro* reflected the pathology of SD.

The SDIA method has been established as a method to efficiently produce neural lineage cells from mouse and primate ES and iPS cells *in vitro* [37]; ES or iPS cells are co-cultured with PA6 stroma cells in serum-free medium, and differentiation into neural lineage cells may be almost selectively induced from ES and iPS cells. We previously reported that when the differentiation of NSC colonies was induced from SD-iPSCs by a co-culture with PA6 stroma cells in serum-free medium, the number and area of colonies were smaller than those in WT-iPSCs [7]. However, we did not confirm whether this abnormal differentiation into NSC colonies reflected the pathology of SD. Thus, we investigated whether abnormal differentiation into NSC colonies observed in SD-iPSCs is reduced by miglustat and *Hexb* gene transfection in SD-iPSCs. Abnormal differentiation was reduced by miglustat. It was also reduced by forced *Hexb* gene transfection in SD-iPSCs. These results clarified that abnormal differentiation into NSC colonies, observed using the SDIA method, reflects the pathology of SD. Furthermore, when NSCs cultured from SD-iPSC-derived NSC colonies were induced to differentiate, NSCs and neurons decreased and astrocytes increased, suggesting that differentiation ability was also promoted in these NSCs. This result was similar to that obtained in neurospheres derived from SD mouse fetal brain and those cultured from SD-iPSC-derived cerebral cortex-like tissue, in which differentiation from NSCs was promoted, which inhibited differentiation into neurons, thereby increasing astrocytes.

The results obtained clarified that the abnormal differentiation of SD-iPSCs toward a neural lineage *in vitro* reflected the pathology of SD. This technique is applicable not only to basic studies, such as those elucidating the pathology of the disease, but also to screening for drug development. For example, novel drugs for SD may be easily searched for and on a large-scale using the area and number of NSC colonies formed employing the SDIA method.

## Conclusions

The number of SD mouse (*Hexb*^−/−^) fetal brain-derived NSCs was reduced and differentiation from NSCs was promoted, which may have inhibited differentiation into neurons and enhanced differentiation into astrocytes. The number of SD-iPSC-derived NSCs was also reduced, similar to that of SD mouse fetal brain-derived neurospheres, and differentiation from NSCs was promoted, which may have inhibited differentiation into neurons and enhanced differentiation into astrocytes. This abnormal differentiation of SD-iPSCs toward a neural lineage was reduced by miglustat, and was also reduced by *Hexb* gene transfection in SD-iPSCs, suggesting that differentiation ability following the time axis is altered in SD mice. The differentiation ability of NSCs is promoted, completing differentiation into neurons earlier, while the timing of differentiation into astrocytes is accelerated. The results obtained clarified that the abnormal differentiation of SD-iPSCs toward a neural lineage observed *in vitro* reflected the pathology of SD, and the *in vitro* system of SD-iPSC differentiation toward a neural lineage may be applicable to the screening of seed compounds of therapeutic drugs for the treatment of SD.

## Supporting information

S1 Fig Cell proliferation in SFEBq-induced cortical tissues of WT-iPSC and SD-iPSC. WT-iPSCs and SD-iPSCs were plated on 96-well low cell-adhesion plates in G-MEM medium supplemented with 7% KSR in the presence or absence of 5 μM miglustat, an inhibitor of the enzyme glucosylceramide synthase, and cultured for 7 days. SFEBq-induced cortical tissues of WT-iPSC and SD-iPSC were dissociated mechanically to single-cell suspensions and replated onto poly-ornithine/fibronectin-coated culture dishes. One hour after plating, proliferating NSCs were determined by using the Click-iT EdU Alexa Fluor 488 Imaging kit (green). Immunostaining of differentiated cells for nestin (red) with DAPI nuclear staining (blue). The scale bar indicates 100 μm.
(TIF)

S2 Fig Proliferation of NSCs isolated from SFEBq-induced cerebral cortical tissues of WT-iPSC and SD-iPSC. Primary neurospheres isolated from the SFEBq-induced cerebral cortical tissues of WT-iPSC and SD-iPSC (with or without miglustat) were dissociated mechanically to single-cell suspensions and replated onto poly-ornithine/fibronectin-coated culture dishes. One hour after plating, proliferating NSCs were determined by using the Click-iT EdU Alexa Fluor 488 Imaging kit (green). Immunostaining of differentiated cells for nestin (red) with DAPI nuclear staining (blue). The scale bar indicates 100 μm.
(TIF)

S3 Fig Cell proliferation in SDIA-induced colonies of WT-iPSC and SD-iPSC. WT-iPSCs and SD-iPSCs were co-cultured on PA6 stromal cells as single cells to form colonies in G-MEM medium supplemented with 10% KSR in the presence or absence of 5μM miglustat, and cultured for 7 days. SDIA-induced colonies of WT-iPSC and SD-iPSC were dissociated mechanically to single-cell suspensions and replated onto poly-ornithine/fibronectin-coated culture dishes. One hour after plating, proliferating NSCs were determined by using the Click-iT EdU Alexa Fluor 488 Imaging kit (green). Immunostaining of differentiated cells for nestin (red) with DAPI nuclear staining (blue). The scale bar indicates 100 μm.
(TIF)

S4 Fig Cell proliferation in neurospheres cultured from SD-iPSC-derived NSC colonies. Primary neurospheres isolated from the SDIA-induced colonies of WT-iPSC and SD-iPSC (with or without miglustat) were dissociated mechanically to single-cell suspensions and replated onto poly-ornithine/fibronectin-coated culture dishes. One hour after plating, proliferating NSCs were determined by using the Click-iT EdU Alexa Fluor 488 Imaging kit (green). Immunostaining of differentiated cells for nestin (red) with DAPI nuclear staining (blue). The scale bar indicates 100 μm.
(TIF)
