# Supplementary figures and images for: Abnormal differentiation of Sandhoff disease model mouse-derived multipotent stem cells toward a neural lineage
Source: PLoS One. 2017 Jun 2;12(6):e0178978. doi: 10.1371/journal.pone.0178978 (PMC5456357; doi:10.1371/journal.pone.0178978)

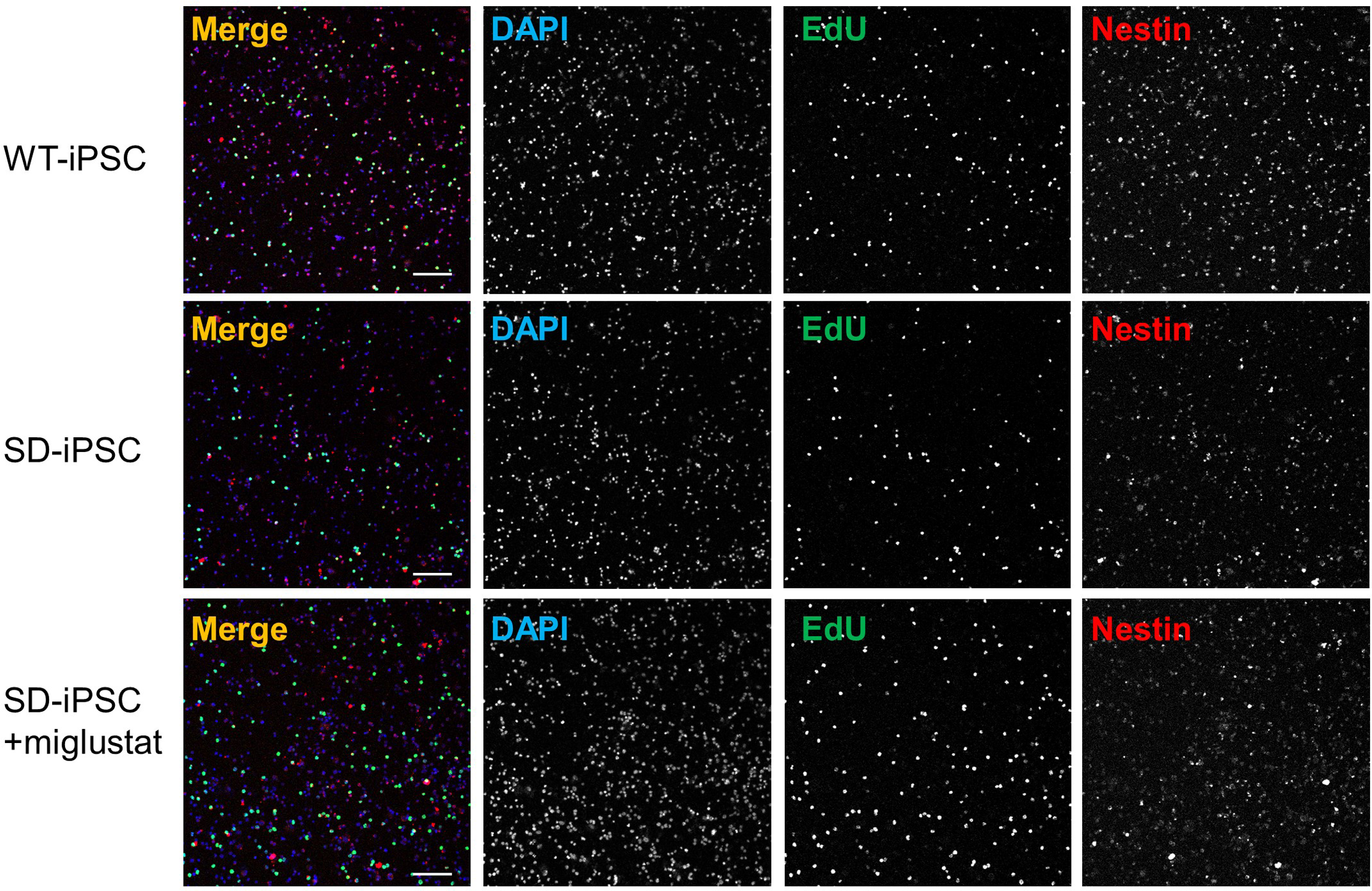

Supplement: S1 Fig — WT-iPSCs and SD-iPSCs were plated on 96-well low cell-adhesion plates in G-MEM medium supplemented with 7% KSR in the presence or absence of 5 μM miglustat, an inhibitor of the enzyme glucosylceramide synthase, and cultured for 7 days. SFEBq-induced cortical tissues of WT-iPSC and SD-iPSC were dissociated mechanically to single-cell suspensions and replated onto poly-ornithine/fibronectin-coated culture dishes. One hour after plating, proliferating NSCs were determined by using the Click-iT EdU Alexa Fluor 488 Imaging kit (green). Immunostaining of differentiated cells for nestin (red) with DAPI nuclear staining (blue). The scale bar indicates 100 μm. (TIF) [file pone.0178978.s001.tif]

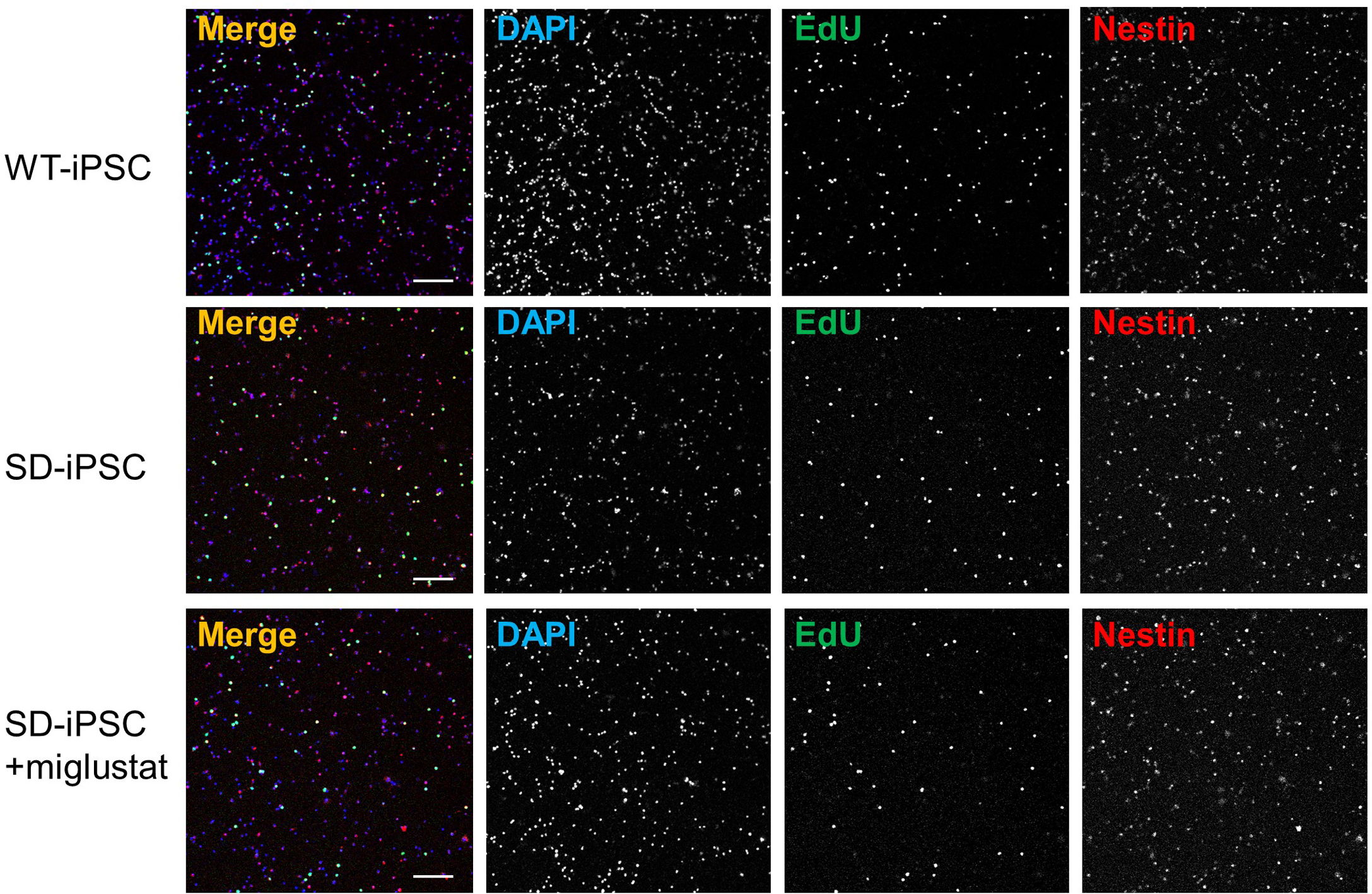

Supplement: S2 Fig — Primary neurospheres isolated from the SFEBq-induced cerebral cortical tissues of WT-iPSC and SD-iPSC (with or without miglustat) were dissociated mechanically to single-cell suspensions and replated onto poly-ornithine/fibronectin-coated culture dishes. One hour after plating, proliferating NSCs were determined by using the Click-iT EdU Alexa Fluor 488 Imaging kit (green). Immunostaining of differentiated cells for nestin (red) with DAPI nuclear staining (blue). The scale bar indicates 100 μm. (TIF) [file pone.0178978.s002.tif]

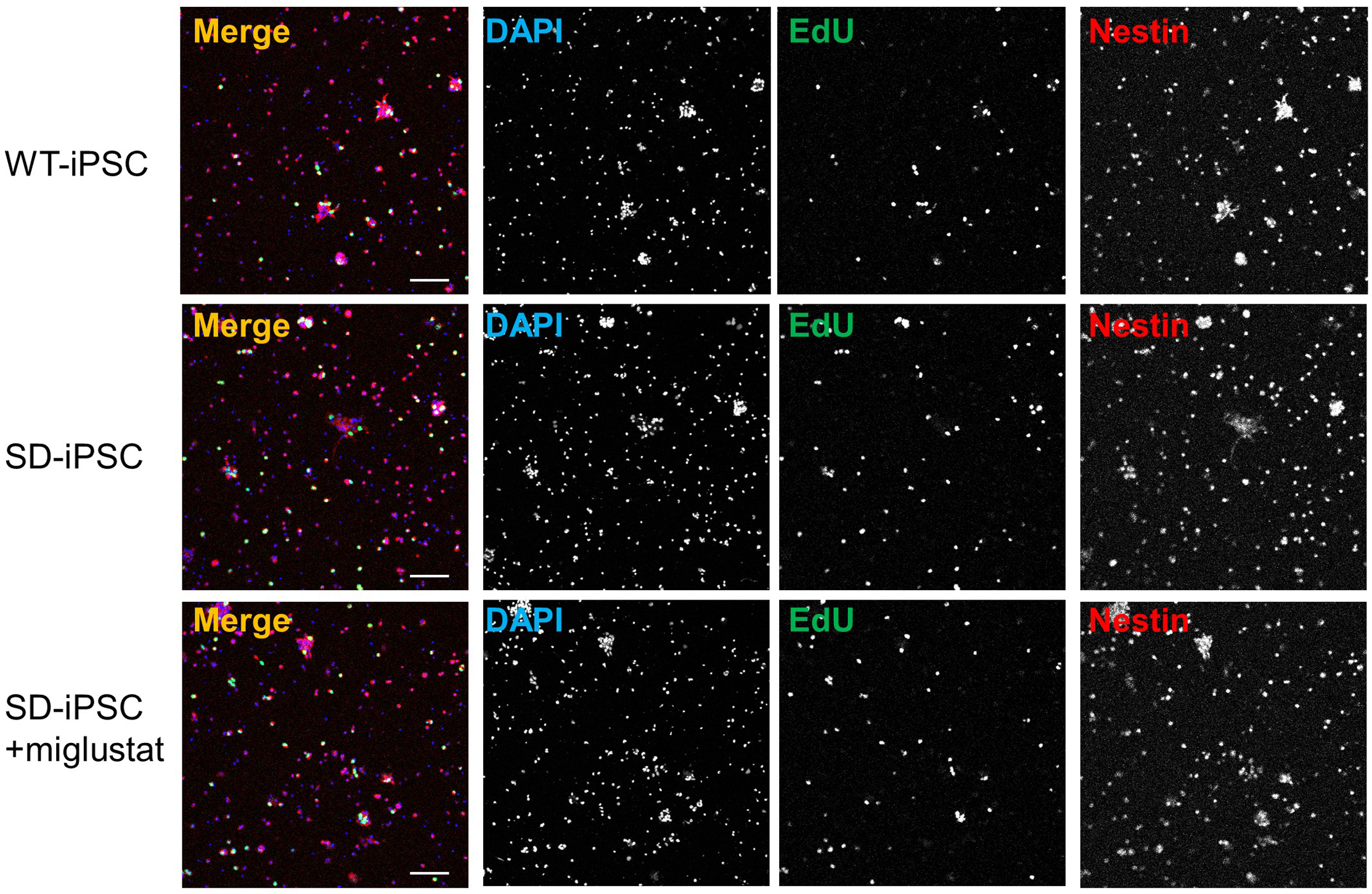

Supplement: S3 Fig — WT-iPSCs and SD-iPSCs were co-cultured on PA6 stromal cells as single cells to form colonies in G-MEM medium supplemented with 10% KSR in the presence or absence of 5μM miglustat, and cultured for 7 days. SDIA-induced colonies of WT-iPSC and SD-iPSC were dissociated mechanically to single-cell suspensions and replated onto poly-ornithine/fibronectin-coated culture dishes. One hour after plating, proliferating NSCs were determined by using the Click-iT EdU Alexa Fluor 488 Imaging kit (green). Immunostaining of differentiated cells for nestin (red) with DAPI nuclear staining (blue). The scale bar indicates 100 μm. (TIF) [file pone.0178978.s003.tif]

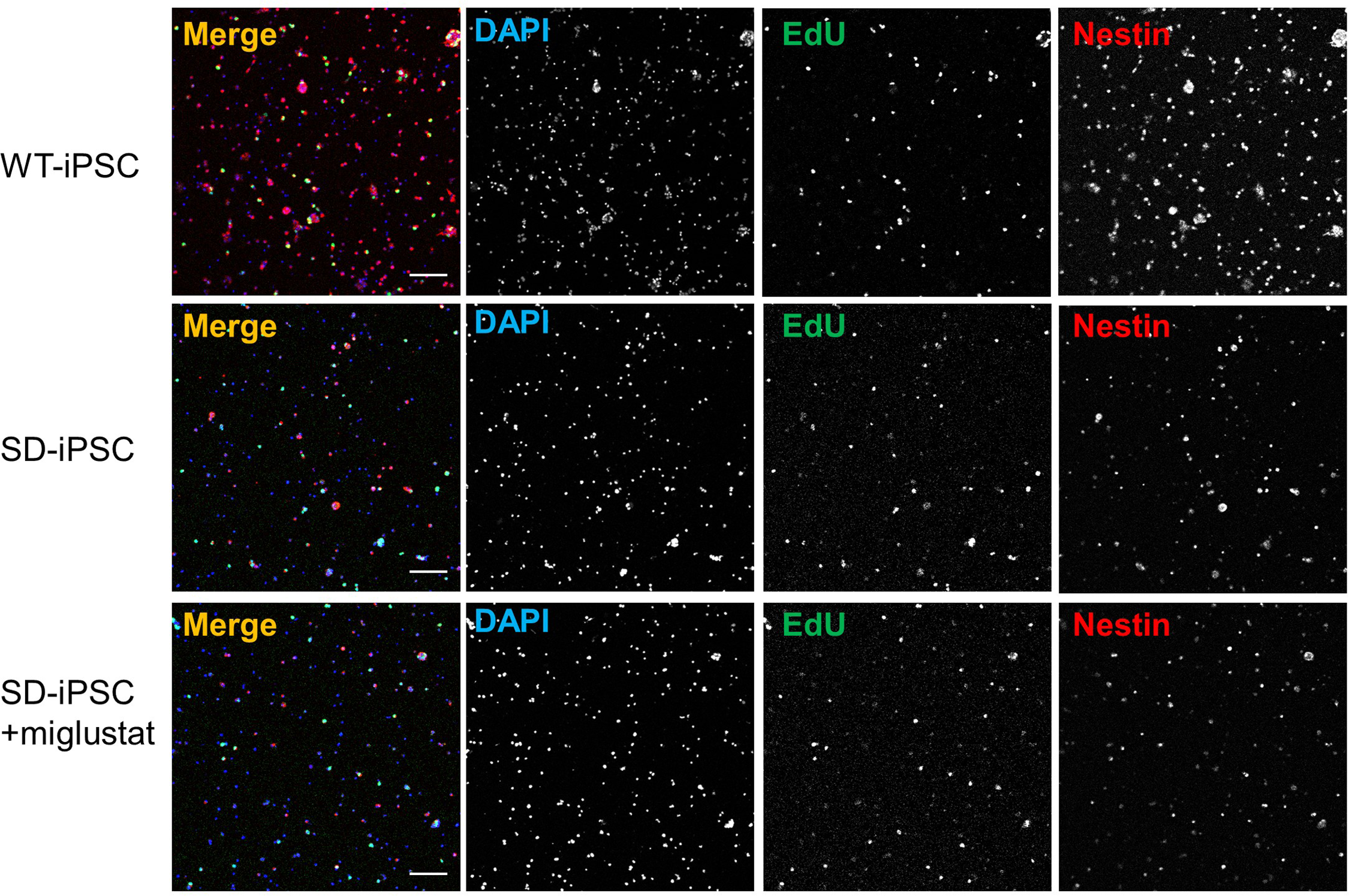

Supplement: S4 Fig — Primary neurospheres isolated from the SDIA-induced colonies of WT-iPSC and SD-iPSC (with or without miglustat) were dissociated mechanically to single-cell suspensions and replated onto poly-ornithine/fibronectin-coated culture dishes. One hour after plating, proliferating NSCs were determined by using the Click-iT EdU Alexa Fluor 488 Imaging kit (green). Immunostaining of differentiated cells for nestin (red) with DAPI nuclear staining (blue). The scale bar indicates 100 μm. (TIF) [file pone.0178978.s004.tif]
